# Cocaine hydrochloride, cocaine methiodide and methylenedioxypyrovalerone (MDPV) cause distinct alterations in the structure and composition of the gut microbiota

**DOI:** 10.1038/s41598-023-40892-1

**Published:** 2023-08-23

**Authors:** Mariana Angoa-Pérez, Branislava Zagorac, Dina M. Francescutti, Zachary D. Shaffer, Kevin R. Theis, Donald M. Kuhn

**Affiliations:** 1 Research and Development Service, John D. Dingell VA Medical Center, Detroit, MI USA; 2 Department of Psychiatry and Behavioral Neurosciences, Wayne State University School of Medicine, Detroit, MI USA; 3 Department of Physiology, Wayne State University School of Medicine, Detroit, MI USA; 4 Department of Biochemistry, Microbiology, and Immunology, Wayne State University School of Medicine, Detroit, MI USA

**Keywords:** Computational biology and bioinformatics, Neuroscience

## Abstract

Cocaine is a highly addictive psychostimulant drug of abuse that constitutes an ongoing public health threat. Emerging research is revealing that numerous peripheral effects of this drug may serve as conditioned stimuli for its central reinforcing properties. The gut microbiota is emerging as one of these peripheral sources of input to cocaine reward. The primary objective of the present study was to determine how cocaine HCl and methylenedioxypyrovalerone, both of which powerfully activate central reward pathways, alter the gut microbiota. Cocaine methiodide, a quaternary derivative of cocaine that does not enter the brain, was included to assess peripheral influences on the gut microbiota. Both cocaine congeners caused significant and similar alterations of the gut microbiota after a 10-day course of treatment. Contrary to expectations, the effects of cocaine HCl and MDPV on the gut microbiota were most dissimilar. Functional predictions of metabolic alterations caused by the treatment drugs reaffirmed that the cocaine congeners were similar whereas MDPV was most dissimilar from the other two drugs and controls. It appears that the monoamine transporters in the gut mediate the effects of the treatment drugs. The effects of the cocaine congeners and MDPV on the gut microbiome may form the basis of interoceptive cues that can influence their abuse properties.

Cocaine is a powerful addictive drug of abuse that remains a significant threat to public health. In addition to its abuse properties, habitual cocaine use is associated with numerous comorbid medical conditions that range in severity from irritation of the nasal septum to increased risk for stroke and seizures^1^, a wide variety of cardiovascular abnormalities^2,3^ and ischemic injury to the gastrointestinal (GI) tract^4^. Chronic cocaine use leads to a vast array of changes in the CNS to include persistent alterations in synaptic properties in neurons of the dopamine (DA) reward pathway^5^, and plasticity changes in excitatory transmission in the nucleus accumbens^6^ and along corticostriatal pathways^7^. These processes likely act in concert through all phases of cocaine abuse disorder to modulate acquisition of self-administration, extinction, craving and relapse. Although numerous targets for therapeutic intervention have emerged, it is generally accepted by experts in the field that an effective treatment for cocaine addiction is lacking^8^.

While emphasis on achieving a better understanding of substance use disorders has naturally focused on intrinsic CNS mechanisms, it is possible that signals from outside of the CNS could play a role in cocaine abuse. While gut microbiota is a target of peripheral action for abused drugs, the mechanisms underlying this interaction remain elusive. The bulk of the microbiota resides in the GI tract and is composed of bacteria, fungi, viruses and archaea. It has been estimated that the human GI system contains > 1000 bacterial species and ~4 × 10^13^ microorganisms (same as number of human cells)^9^, and gut microbiota express ~100 times as many unique genes as the host human genome^10,11^. Normal functioning of the gut microbiota is essential to the maintenance of human health. An imbalance in the microbiota composition (i.e., dysbiosis) has been linked to numerous diseases including cancer, diabetes, obesity, immune dysfunction and inflammatory bowel disease^12,13^. It is also emerging that gut microbiota dysbiosis can play a role in numerous neurological (e.g., Parkinson’s disease, Alzheimer’s disease^14^) and psychiatric disorders (e.g., autism^15^, and depression and anxiety^16^).

With regard to drugs of abuse, a small but growing literature is implicating the gut microbiota in alcohol abuse and withdrawal^17–19^, nicotine and smoking^20,21^ and in methamphetamine-induced conditioned place preference (CPP)^22^. The prevention of diet-induced obesity by ∆^9^-THC induced has been linked to its actions on the microbiota^23^. Despite the paucity of published papers in the area of drug abuse and gut microbiota interactions, the premise for undertaking such studies with regard to cocaine is actually quite compelling. Emerging results have shown that cocaine causes dysbiosis in the gut microbiota of humans^24^ and rodents^25,26^ and that dysbiosis affects responses to cocaine^27^. Depletion of gut bacteria by treatment with a prolonged course of antibiotics increased sensitivity to cocaine CPP and enhanced its locomotor stimulating properties^28^. The report by Buch and colleagues^26^ also documented cocaine-induced upregulation of proinflammatory mediators in the gut along with a compromise of mucosal barrier integrity. In light of the fact that bowel ischemia is a major side effect of cocaine abuse in humans^4^, it is relevant that modulation of the gut microbiota can prevent intestinal ischemia/reperfusion injury^29,30^. The current status of gut microbiota roles in substance use disorders has been reviewed recently^31,32^.

In the present study, we report the effects of cocaine hydrochloride (HCl), cocaine methiodide (MI) and methylenedioxypyrovalerone (MDPV) on the gut microbiota. Cocaine HCl was chosen for this study to broaden the understanding of its actions on the gut microbiota and for comparison to its MI analog. The rewarding and reinforcing effects of cocaine have all been primarily associated with its blockade of the DA transporter (DAT) and the resulting elevation in extracellular DA levels^33^. Cocaine MI is a quaternary derivative of cocaine that cannot cross the blood–brain barrier^34^ and may differentiate the peripheral and central effects that contribute to the rewarding effects of cocaine HCl^35^. MDPV was included because it shares many of the rewarding properties of cocaine HCl based on its ability to support self-administration^36–38^, form a CPP^39–41^ and facilitate intracranial self-stimulation^42–44^. MDPV is also a more potent and efficacious inhibitor of the DAT than cocaine HCl^45^. Therefore, this study sheds light on the mechanisms by which cocaine can affect the gut microbiome by pointing the role of monoamine transporters differentially activated by cocaine and analog drugs in the gut, and it also contributes to understand the crosstalk between gut microbiome and brain axis in responses to cocaine. This was possible through the use of cocaine methiodide, which does not cross the blood-brain barrier, limiting its effects to peripheral targets.

## Materials and methods

### Study drugs

Cocaine MI and MDPV were provided by the NIDA Research Resources Drug Supply Program. Cocaine HCl was purchased from Sigma-Aldrich (St. Louis, MO, USA).

### Animals and drug treatment

Female C57BL/6 mice (Envigo, Indianapolis, IN, USA) weighing 18–25 g at the time of experimentation were initially housed 6–7 per cage in large shoe-box cages in a room with constant temperature and humidity and with alternating 12 h periods of light and darkness. Females were used in order to maintain consistency with our previous study that examined effects of synthetic psychoactive cathinone drugs and amphetamine stimulants on the gut microbiota^46^. In addition, studies in humans and rodents indicate that females are more vulnerable to the reinforcing effects of cocaine^47^. Given that sex hormones can drive differences in gut microbiome, a stand-alone sex comparison is more suitable as an independent and subsequent study^48^. Mice had free access to food and water. Diet consisted of standard laboratory rodent chow (LabDiet 5001) containing 28.5% protein, 13.5% fat, and 58% carbohydrates. All mice used in these studies were from the same cohort. The mice used were randomly divided into treatment groups (N = 6–7 mice per group) and were injected intraperitoneally (i.p.) once daily for 10 days with saline (controls), cocaine HCl, cocaine MI or MDPV. Doses of the study drugs were chosen from published accounts showing self-administration for cocaine HCl and MDPV and are based on average daily drug intake achieved for each drug^36,49^. Doses were estimated to be equipotent for self-administration for cocaine HCl (20 mg/kg) and MDPV (2.0 mg/kg). The dose of cocaine MI (26 mg/kg) was equimolar to that of cocaine HCl (20 mg/kg). This treatment regimen is similar to the one used for cocaine HCl treatment of mice by Chivero and colleagues in their recent paper^26^. Additionally, we are modeling repeated cocaine use in a similar time frame used in the context of well-established paradigms modeling cocaine addiction-like behaviors such as CPP and behavioral sensitization^28,50^*.* Mice were sacrificed 2 days after the final treatment with each study drug. After sacrifice, caecum contents were harvested, weighed and stored frozen at − 80 °C until DNA isolation. Stressors such as noise and handling by multiple persons were avoided. Mice were monitored daily for signs of distress or injury by visualization of general parameters such as separation from the group, decreased grooming, piloerection, abnormal posture (e.g., hunching), and changes in behavior while being handled (e.g., placid or more aggressive), until the experimental endpoint. The Institutional Care and Use Committee of Wayne State University approved the animal care and experimental procedures (IACUC # 19-02-0973). All procedures were also in compliance with the NIH *Guide for the Care and Use of Laboratory Animals* and were conducted in compliance with ARRIVE guidelines and under IACUC-approved protocols.

### Microbiota analysis

DNA was extracted from caecum contents (~200 mg wet weight) using QIAamp PowerFecal DNA kits and sample DNA concentrations were determined using a Qubit 4 Fluorometer (range 70–100 ng/µl). Samples were sequenced in duplicate on an Illumina MiSeq system using a 2 × 250 cycle V2 kit with Illumina reagents and Illumina sequencing procedures detailed by Kozich and colleagues^51^. The 16S rRNA gene primers targeted the V4 region of the gene (forward primer: 5′-GTGCCAGCMGCCGCGGTAA-3′; reverse primer: 5′-GGACTACHVGGGTWTCTAAT-3′). The 16S rRNA gene sequences from the paired fastq files were processed with the Divisive Amplicon Denoising Algorithm (DADA2) pipeline to obtain merged, denoised, chimera-free, inferred amplicon sequence variants (ASVs) as previously reported by our laboratory^52^. The ASVs were classified taxonomically using the Silva reference database (v132) and the bacterial community data was thereafter visualized and statistically analyzed using PAST software (v3.20^53^). Microbiota diversity was characterized in terms of α-diversity using the Chao1 (i.e., community richness) and Shannon and Simpson (1-D) (i.e., community heterogeneity) indices. β-diversity was assessed using the Jaccard (i.e., shared composition) and Bray–Curtis (i.e., shared structure) similarity indices based on relative abundance data. High-dimensional class comparisons were carried out with linear discriminant analysis effect size (LEfSe) in an online interface^54^ using default parameters with the exception that LDA score was set to 3.6. Heat maps were generated using MetaboAnalyst 4.0^55^.

### Inferred functional analysis

The PICRUST 2 software package^56^ was used for predicting functional abundances based on marker gene sequences (16S rRNA sequencing data). Statistical analysis of metagenomic profiles (STAMP) was used for analysis. MetaCyc ontology predictions^57^ were used for metabolic pathways classification.

### Data analysis and statistics

The indices for α-diversity were obtained using PAST software. The results were analyzed statistically with a one-way ANOVA, and subsequent post hoc comparisons were performed with Tukey’s test using GraphPad Prism (v6.07) for Windows (GraphPad Software, La Jolla, CA, USA, www.graphpad.com). The indices for β-diversity were also calculated, and statistical analyses were carried out, using PAST software. The results were analyzed using one-way NPMANOVAs. Taxonomic distributions at the phylum level were analyzed with a mixed-effects model (fixed effects of treatment X phylum) controlled for multiple comparisons using Benjamini–Hochberg correction. Lower taxonomic levels (treatment X taxa) were analyzed with a two-way ANOVA followed by post hoc comparisons using Tukey’s tests in GraphPad Prism. Analysis of metagenomic profiles with STAMP was done with ANOVA and Tukey–Kramer post-hoc tests.

## Results

### Effects of treatment drugs on the gut microbiota at the ASV level

**Figure. 1:**
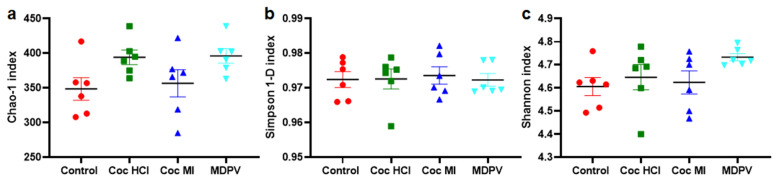
Effects of coc HCl, coc MI and MDPV on α-diversity. Data are presented as mean ± SEM for Chao-1 (**a**), Simpson 1-D (**b**) and Shannon (**c**) indices. N = 6 per group.

Samples were analyzed with 3 different α-diversity indices: Chao-1 (Fig. 1a), Simpson 1-D (Fig. 1b) and Shannon (Fig. 1c) to account for specific biases. Chao-1 is a richness estimator, whereas Simpson and Shannon are two different measures of richness and evenness of the microbial composition^58^. Simpson's complementary diversity (1-D) index relies on Simpson's dominance (D), which indicates the species dominance^58^. Thus, greater values of Simpson 1-D indicate increases in diversity. The Shannon index places a greater weight on species richness and its value increases as both the number of species and their evenness increase^58^. An analysis of the effects of cocaine HCl, cocaine MI and MDPV on α-diversity (Fig. 1) shows that none of the drugs changed α-diversity by comparison to controls.

**Figure 2:**
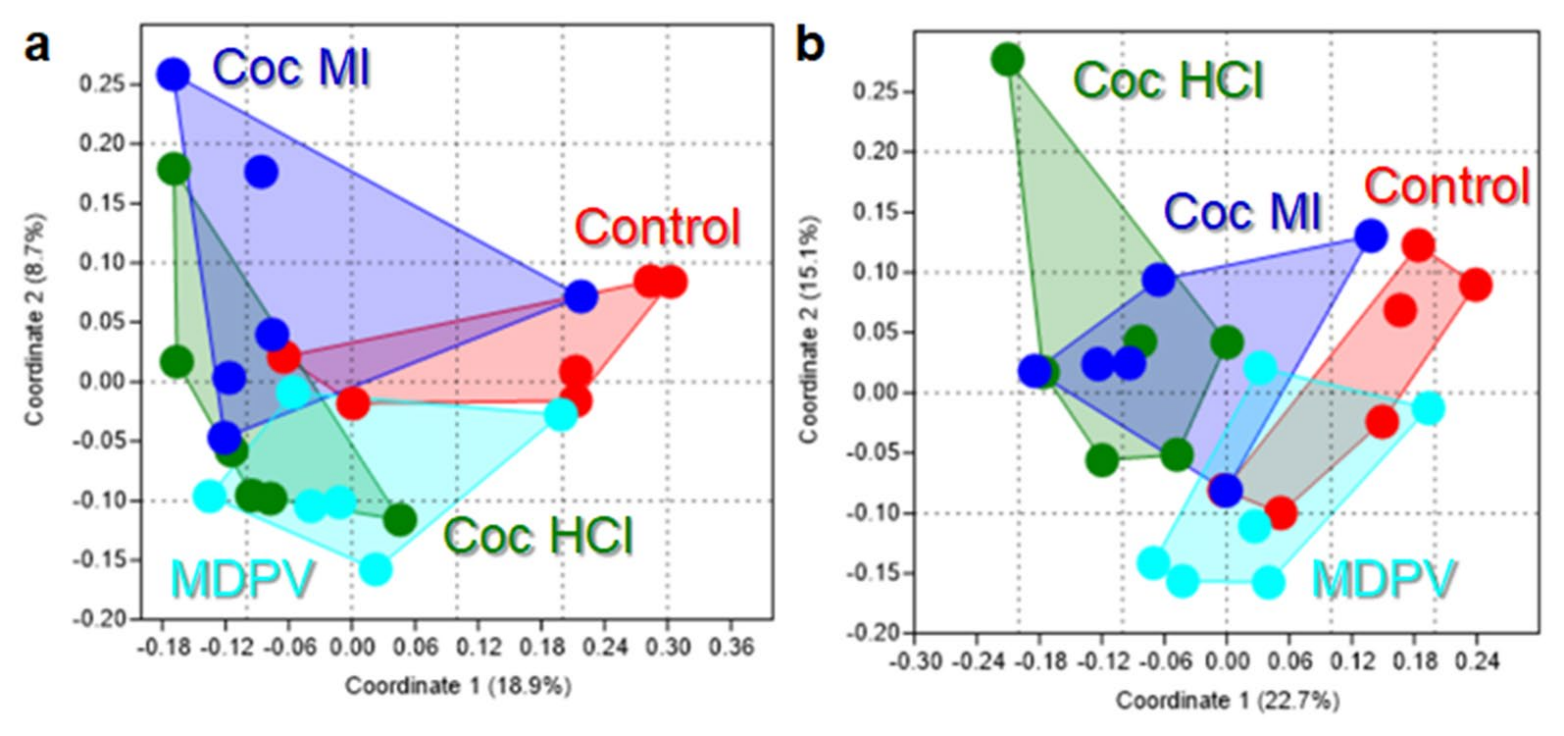
Effects of coc HCl, coc MI and MDPV on β-diversity. Principal Coordinates Analyses (PCoA) illustrating differences in the shared composition (i.e., Jaccard index; **a**) and the shared structure (i.e. Bray–Curtis index, **b**) of gut microbiome profiles among mice treated with the different study drugs. All pairwise statistical comparisons are included in Supplementary Table S1.

With respect to β-diversity, analyses using the Jaccard index, which reflects shared microbiota membership (i.e., community composition), indicated that the ASV profiles of the study drugs clustered together and apart from one another as shown in Fig. 2a. The main effect of treatment drug was significant (p = 0.0008, NPMANOVA). Post hoc analysis revealed that all pairwise comparisons between drugs were significantly different with the exception of cocaine HCl versus cocaine MI. Analysis of β-diversity using the Bray–Curtis index, which reflects overall microbiota structure (i.e., not just membership) agreed well with the Jaccard analysis showing distinct clustering of the ASV profiles for each drug (Fig. 2b). The main effect of treatment drug was significant (p = 0.0001, NPMANOVA). All pairwise comparisons between study drugs were statistically significant in the Bray–Curtis analysis. The results of these pairwise statistical tests for β-diversity are presented in Supplementary Table S1.

**Figure 3:**
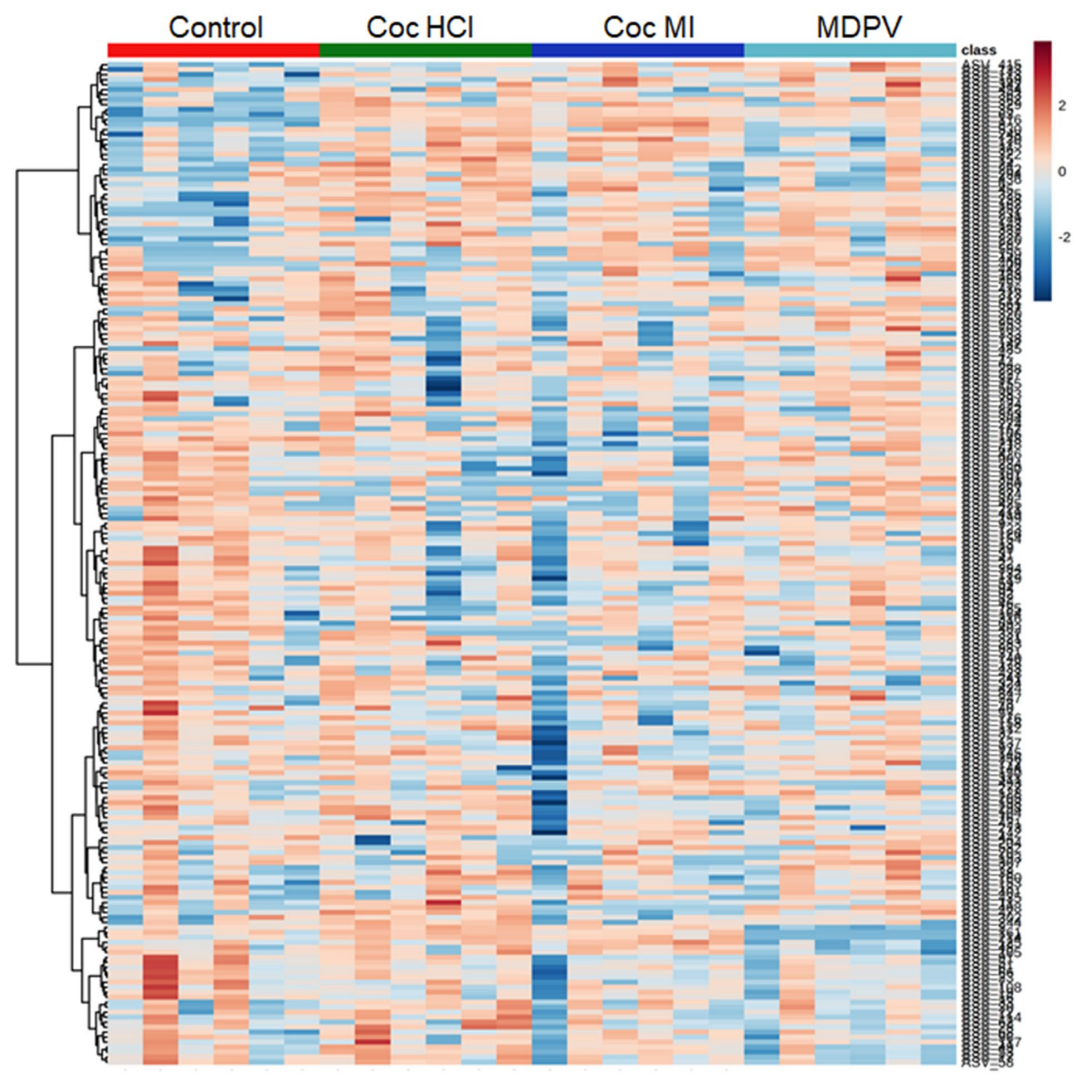
Heat map illustrating the relative abundances of ASVs after treatment with coc HCl, coc MI and MDPV. The most prominent ASVs (≥ 3% average relative abundance) among treatment groups are plotted for each drug. All subjects in each treatment group are arrayed in columns and bacterial taxonomies are arrayed in rows. Taxonomic clustering was done using the Ward algorithm.

The ASV profiles (≥ 3% relative abundance) varied substantially among the treatment drugs as shown in the heat map depicting individual subjects in the color-coded columns shown in Fig. 3. The treatment drugs caused changes in percent relative abundance across the represented phylogenetic tree (i.e., viewed vertically on the heat map). Cocaine HCl and cocaine MI were very similar (with the exception of one outlier in the cocaine MI group) to each other and differed from controls and the MDPV group. Both cocaine groups showed higher relative abundances of those taxa with greater overall percent relative abundance and lower relative abundances of taxa with overall lower percent relative abundance. The ASV profiles of MDPV, on the other hand, were different from controls and both cocaine groups showing decreased abundance of taxa with overall intermediate and lower relative abundance and increased abundance of taxa with overall higher relative abundance.

**Figure 4:**
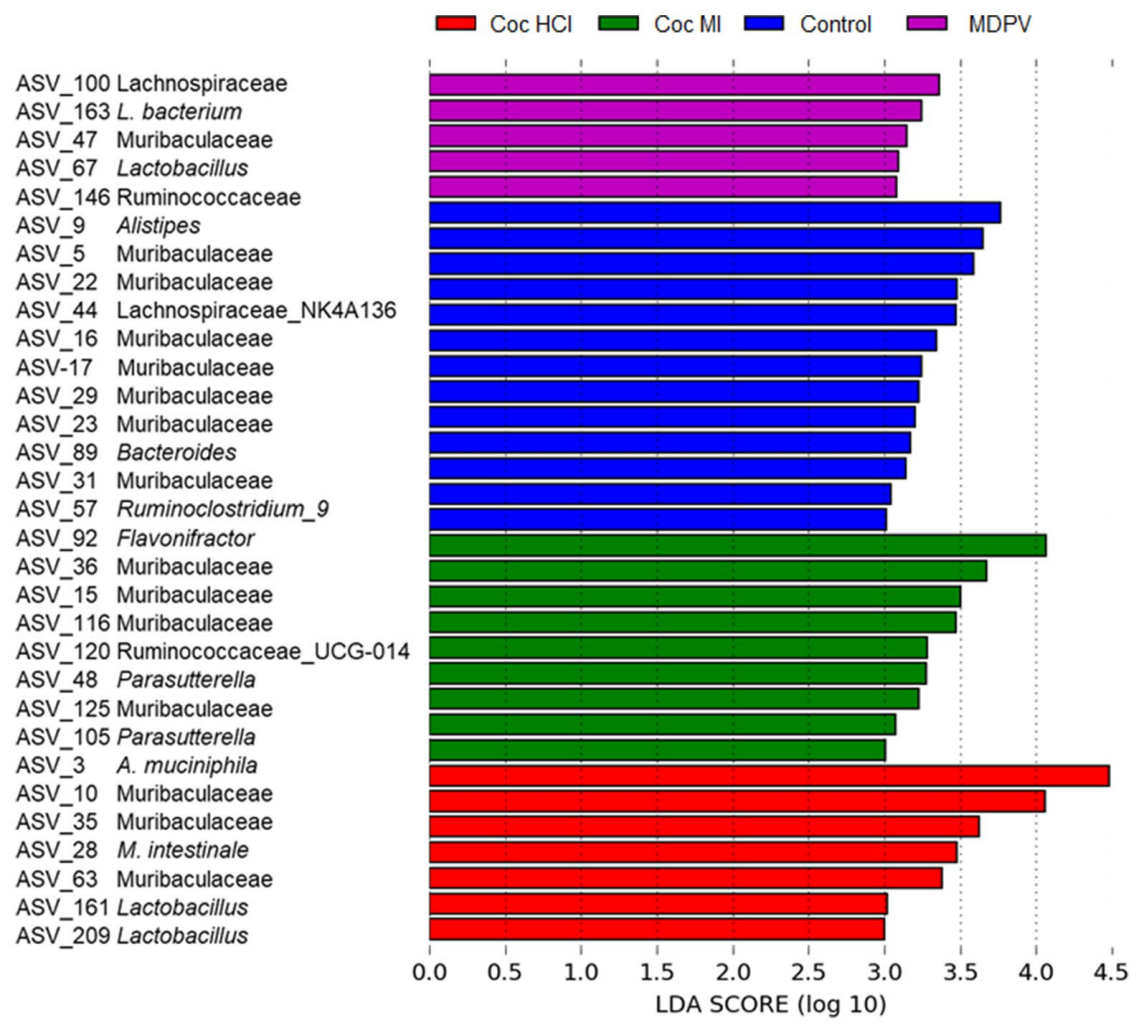
Bacterial taxa that were differentially abundant across treatments with coc HCl, coc MI and MDPV. Linear discriminant analysis effect size (LEfSe) was carried out and the results are presented for taxa with LDA scores of > 3.0 for the treatment groups.

Figure 4 presents results from LEfSe analyses and highlights the effect size (LDA scores > 3.0) of the study drug treatments on affected taxa. LEfSe determines the features (e.g., ASVs) most likely to explain differences between experimental groups by coupling standard tests for statistical significance with additional tests encoding biological consistency and effect relevance^54^. The discriminant taxa for all drugs spanned several phyla but were primarily located within Bacteroidetes and Firmicutes. The greatest number of markers were found for the control group (N=13) followed in number by cocaine MI (N=9) and cocaine HCl (N=7) followed by MDPV with the fewest (N=5). In an attempt to gain more insight into the taxa enriched in each treatment group, all ASVs emerging from the LEfSe analysis were submitted to BLAST analysis and those bacterial species identified with > 99.5% sequence identity are presented in Table 1. Based on the outcome of the LEfSe analyses, species identified in the BLAST searches as determinant for cocaine HCl (6 of 15) whereas those for controls (N=4), cocaine MI (N=3) and MDPV (N=2) were fewer. In general, the species linked to cocaine HCl (e.g., *Akkermansia muciniphila, Muribaculum intestinale*, and 3 strains of Lactobacillus) and cocaine MI (e.g., *Duncaniella muris, Fecalibaculum rodentium, Parabacteroides goldsteinii*) are considered beneficial to the host, and many are associated with anti-inflammatory actions in the gut. Species determinant for controls (e.g., *Sangeribacter muris, Phocaeicola dorei, Flintibacter butyricus*) and MDPV (e.g., *Lactobacillus, faecis*) are short-chain fatty acids and lactic acid producers and contribute to normal GI function.

**Table 1:** Identification by BLAST analysis of bacterial species identified in LEfSe as specific for each treatment group as compared to all others.

ASV #	Bacterial species	% Identity	Treatment group
ASV_5	*Sangeribacter muris*	100	Control
ASV_89	*Phocaeicola dorei*	100	Control
ASV_57	*Flintibacter butyricus*	100	Control
ASV_3	*Akkermansia muciniphila*	100	Coc HCl
ASV_10	*Muribaculaceae bacterium*	100	Coc HCl
ASV_28	*Muribaculum intestinale*	100	Coc HCl
ASV_161	*Lactobacillus johnsonii*	100	Coc HCl
ASV_209	*Lactobacillus reuteri*	100	Coc HCl
ASV-214	*Lactobacillus intestinalis*	100	Coc HCl
ASV_36	*Muribaculum sp. TLL-A4*	100	Coc MI
ASV-15	*Duncaniella muris*	100	Coc MI
ASV_105	*Turicimonas muris*	100	Coc MI
ASV_67	*Lactobacillus faecis*	100	MDPV

*Coc HCl* cocaine HCl, *Coc MI* cocaine MI, *MDPV* Methylenedioxypyrovalerone.

### Effects of treatment drugs on the gut microbiota at the phylum level

**Figure 5:**
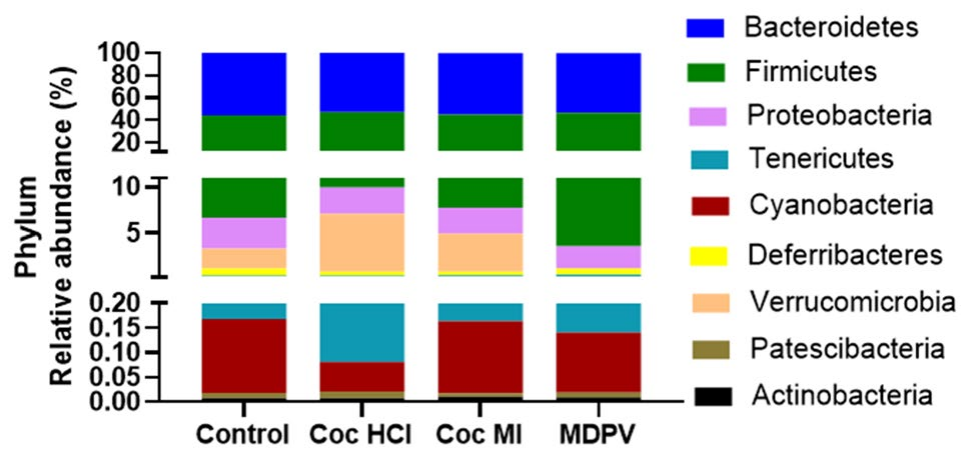
Relative abundances of the 7 most prominent bacterial phyla after treatment with coc HCl, coc MI and MDPV. Results are presented as % relative abundance of each phylum for each study drug. Stacked columns for the mean values for each phylum are included for each of the treatments and controls.

Figure 5 shows the effect of treatments at the phylum level. The effect of treatment drug was not significant whereas the effect of phylum (F (1.38, 30.91) = 2518, p < 0.0001) and the treatment drug ×phylum interaction (F (24, 179) = 3.328, p < 0.0001) were. Multiple comparisons corrected with the Benjamini–Hochberg method revealed that significant drug-induced alterations occurred in the phylum Verrucomicrobia. For this phylum, every drug differed from controls and from each other, with the exception of the comparison between cocaine HCl and Cocaine MI. Both Cocaine HCl and cocaine MI significantly increased the relative abundance of Verucomicrobia by comparison to controls (p < 0.05 for both drugs). Conversely, MDPV caused a significant reduction of Verrucomicrobia compared to controls (p < 0.01). MDPV also reduced the relative abundance of Verrucomicrobia compared to both cocaine HCl (p < 0.05) and cocaine MI (p < 0.001).

### Effects of treatment drugs on taxa at the level of family

**Figure 6:**
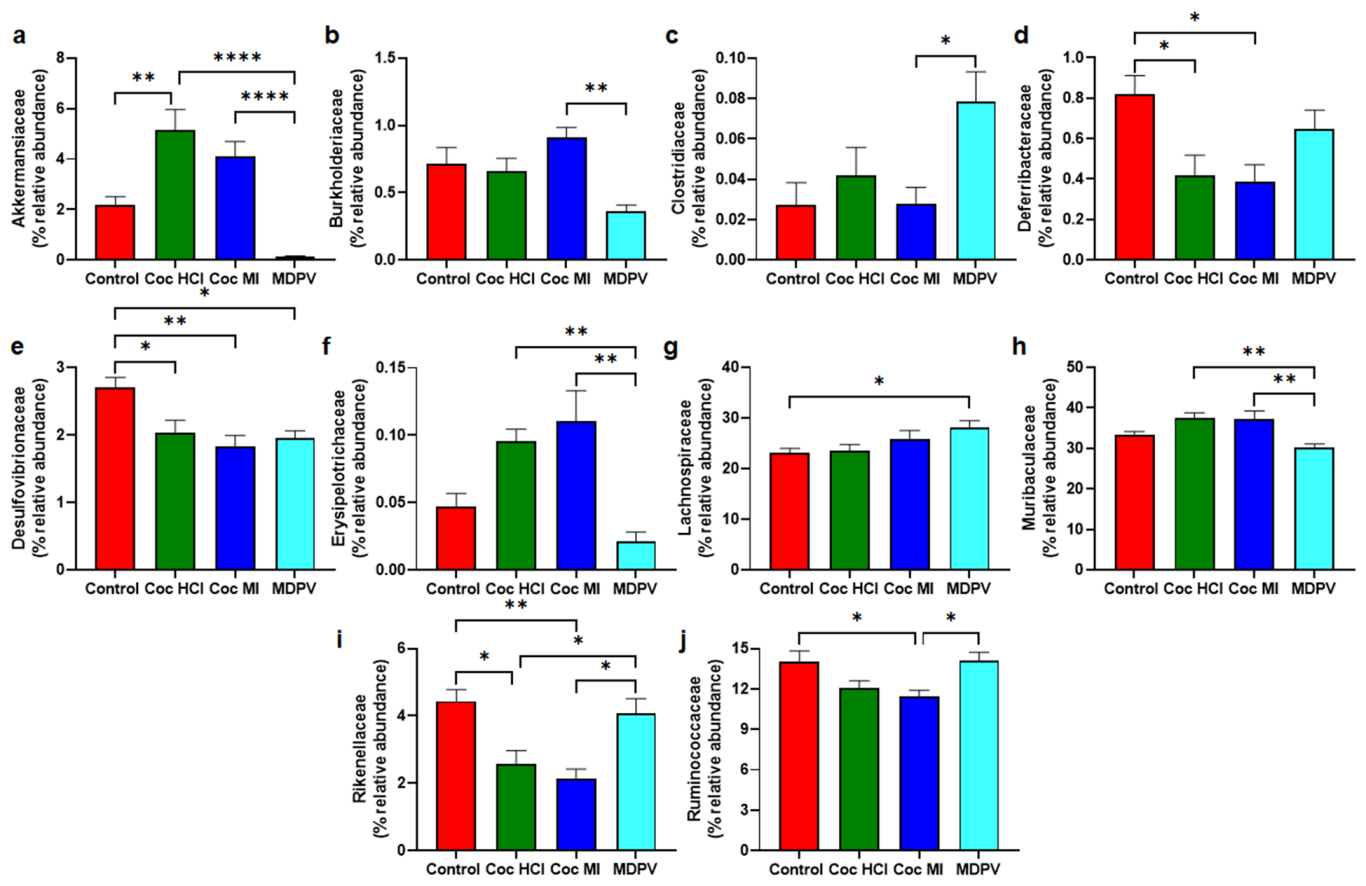
Effects of coc HCl, coc MI and MDPV on selected taxa at the level of family. Results are presented as % relative abundance (mean + SEM) for Akkermansiaceae (**a**), Burkholderlaceae (**b**), Clostridiaceae (**c**), Deferribacteraceae (**d**), Desulfovibrionaceae (**e**), Erysipelotrichaceae (**f**), Lachnospiraceae (**g**), Muribaculaceae (**h**), Rikenellaceae (**i**) and Ruminococcaceae (**j**) for each treatment group. *p < 0.05, **p < 0.01, and ****p < 0.0001 for the comparisons demarked by connecting lines above the bars.

The treatment drugs caused complex changes in the percent relative abundance at taxonomic levels of family in a manner that was largely drug specific. First, the phylum within which most drug-induced changes occurred was Firmicutes (4 of 10 panels of Fig. 6) showing significant alterations in percent relative abundance) followed by two in Bacteroidetes and one each in Proteobacteria, Verrucomicrobia, Deferribacteres, and Thermodesulfobacteriota**.** The greatest number of drug-induced changes from control were seen after cocaine HCl and cocaine MI treatments where both drugs caused significant decreases in families Deferribacteraceae (Fig. 6d), Desulfovibrionaceae (Fig. 6e) and Rikenellaceae (Fig. 6i), while causing increases in family Akkermansiaceae (Fig. 6a). Cocaine MI alone significantly reduced the % relative abundance of Ruminococcaceae (Fig. 6j) while MDPV was associated with decreases in Desulfovibrionaceae (Fig. 6g) and Akkermansiaceae (Fig. 6a) and increases in Lachnospiraceae (Fig. 6g).

### Metabolic and functional predictions

**Figure 7:**
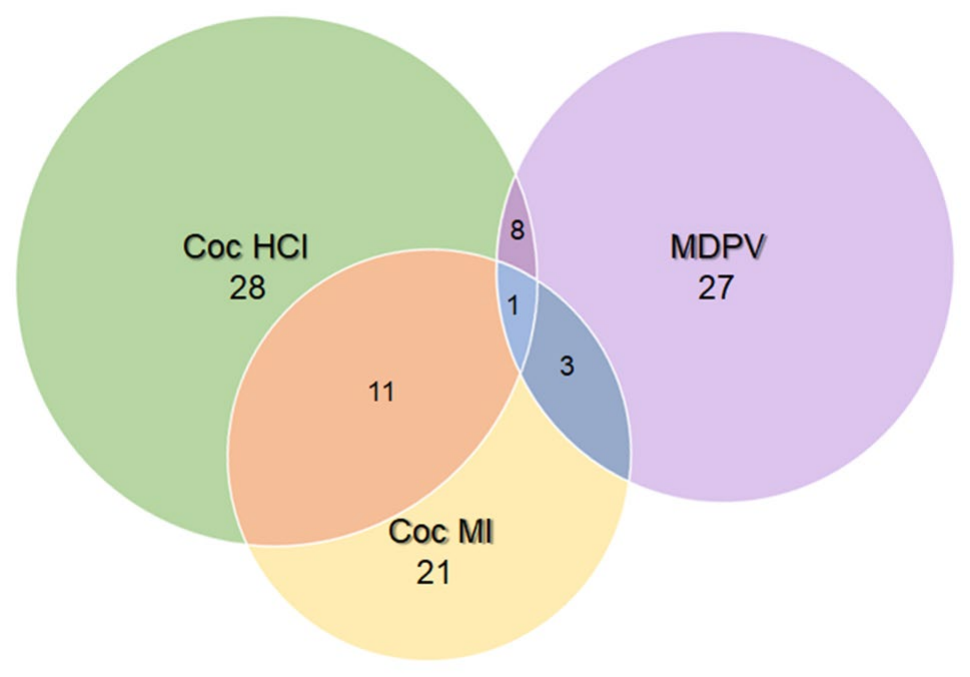
Venn diagram showing the metabolic pathway alterations resulting from coc HCl, Coc MI and MDPV treatment. The number of metabolic pathways that differed significantly from controls for each treatment drug are represented by the major circles. The intersections show the number of pathway alterations shared by two or three of the treatment drugs.

**Figure 8:**
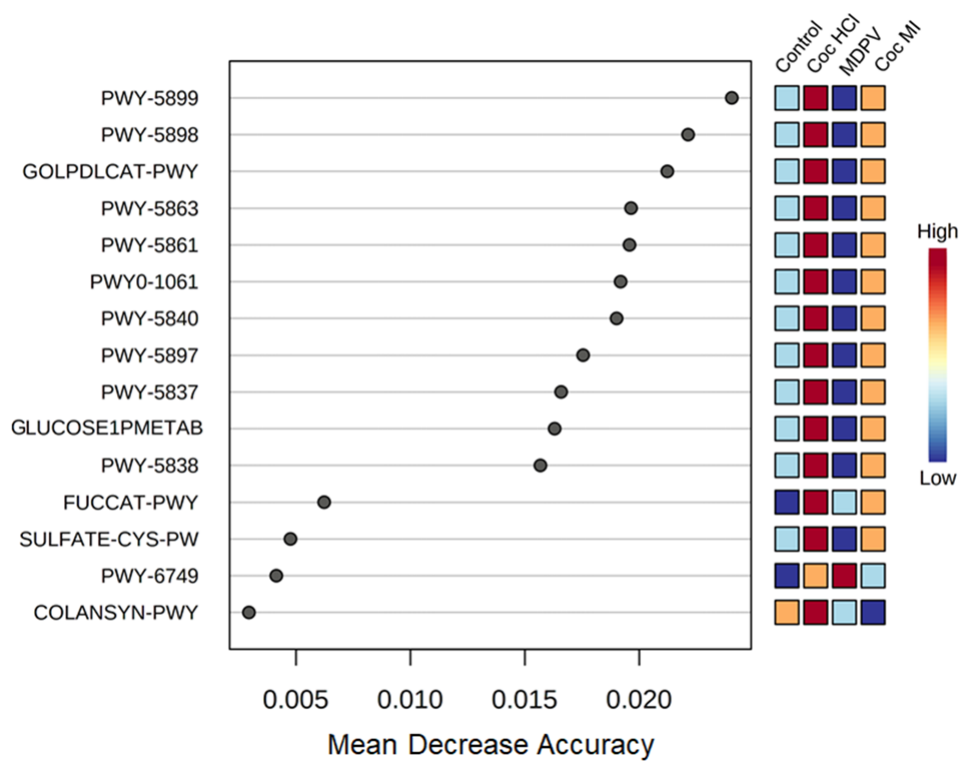
Random forest variable importance plot of the metabolic pathway alterations caused by coc HCl, coc MI and MDPV. Data for the pathway alterations by the treatment drugs is plotted versus mean decrease in accuracy. Higher values for mean decrease in accuracy indicates the importance of a pathway in predicting its association with a treatment drug.

PICRUST 2^56^ was used for predicting metabolic and functional pathway abundances within the gut microbiota based on the 16S rRNA sequencing data. The significant drug-induced effects were analyzed with STAMP and displayed in MetaCyc, a database that includes a collection of metabolic pathways and enzymes from a wide variety of microorganisms and plants. A summary analysis of these data revealed that 76 microbial pathways were modified significantly by one or more of the drug treatments used. Analysis of these MetaCyc pathways revealed that the greatest number of drug-induced changes occurred in the nucleotide metabolism pathway (N = 20) followed closely by the cofactor, carrier and vitamin metabolism pathway (N = 18) and the carbohydrate metabolism pathway (N = 12). A total of 8 alterations occurred in the amino acid metabolism pathway, with fewer changes in the pathways for glycan biosynthesis (N = 4), metabolism of terpenoids (N = 3), energy metabolism (N = 3) and amine and polyamine metabolism (N = 1). All of the predicted drug-induced metabolic changes are presented in Supplementary Table S2. The number of metabolic pathways changed by each treatment is presented as a Venn diagram in Fig. 7. It can be seen that cocaine HCl (N = 28) and MDPV (N = 27) were associated with the highest number of predicted metabolic changes with the fewest associated with cocaine MI (N = 21). The cocaine congeners shared the highest number of pathway alterations (N = 11) while fewer numbers were shared by MDPV and cocaine MI (N = 3) and MDPV and cocaine HCl (N = 8). A single pathway was shared among all three drugs. It is interesting that when cocaine HCl and cocaine MI altered the same metabolic pathway, the direction of alteration was always the same and these comparisons are included in Supplementary Table S3. On the other hand, when cocaine HCl and MDPV caused changes in the same metabolic pathway, the direction of the change was always in opposite directions. If MDPV caused greater changes in a pathway versus cocaine HCl, it was also always greater than cocaine MI and if cocaine HCl > MDPV, cocaine MI was also > than MDPV for that same pathway (Supplementary Table S3). These findings further reinforce other observations that cocaine HCl and cocaine MI were most similar and MDPV was most dissimilar by comparison to the cocaine congeners. Finally, a random forest analysis was carried out to define those metabolic features that allowed discrimination among the drug treatment groups and the results are presented in Fig. 8. Based on mean decrease in accuracy analysis, 11 pathways emerged that discriminate among the 3 drugs and controls, most of which (8 of 11) were in the major MetaCyc pathway of metabolism of cofactors and vitamins. The remaining classifiers fell within pathways of lipid metabolism and carbohydrate metabolism. The three most influential features were PWY-5899 (menaquinol-13 biosynthesis), PWY-5898 (menaquinol-12 biosynthesis) and GOLPDLCAT-PWY (glycerol degradation to 1,3-propanediol). The top 11 pathways most accurately predict the magnitude of the metabolic activation in the order of coc HCl > coc MI > control > MDPV.

## Discussion

The goal of the present study was to compare the effects of cocaine HCl, cocaine MI and MDPV on the gut microbiota. Predictions of how these drugs could alter the gut microbiome depend on whether these drugs are acting directly at the level of the gut or via the brain-to-gut axis. In the former possibility, it would be predicted that cocaine HCl would cause effects similar to those of cocaine MI, based on their sequence similarity, and MDPV would differ from both cocaine congeners. In the latter possibility, it would be predicted that cocaine HCl and MDPV would be most similar in their effects on the gut microbiome in light of the extensive overlap these drugs have on DA reward circuits in the CNS. These include the ability to support self-administration^36–38^, formation of a CPP^39–41^ and enhancement of intracranial self-stimulation^42–44^. MDPV, like cocaine HCl, is a potent blocker of the DAT^45^, a critical mechanism in the addictive actions of these psychostimulants. Cocaine MI, which does not reach the CNS, would be very different from cocaine HCl and MDPV. Taken together, the results suggest direct actions on the gut in light of the similarities between cocaine HCl and cocaine MI, and the dissimilarity of MDPV with the cocaine congeners.

The phyla most altered by the study drugs were Verrucomicrobia and Firmicutes. The alterations that were significantly different from control were increases by cocaine HCl for Verrucomicrobia and increases by MDPV for Firmicutes. MDPV significantly reduced the relative abundance of Verrucomicrobia by comparison to the cocaine congeners and significantly increased the relative abundance of Firmicutes versus both cocaine compounds. At the taxonomic level of family, the drug-induced changes were complex and the majority of the changes were in families within the Firmicutes (N=4) and Bacteroidetes (N=2) phyla. It is interesting that the differences between the cocaine congeners and MDPV remained evident in the alterations of the family taxa with cocaine HCl and cocaine MI changing in opposite directions from those caused by MDPV. For instance, MDPV was significantly lower in abundance of Akkermansiaceae by comparison to the cocaine drugs whereas MDPV was significantly higher in abundance in the Rikenellaceae family by comparison to the cocaine drugs. The only family in which all three drugs had the same effect was in Desulfovibrionaceae where abundance was significantly reduced by comparison to controls. The present findings generally agree with prior studies reporting cocaine-induced dysbiosis in that α-diversity is not changed^25,26^ whereas β-diversity shows significant microbial dissimilarities between cocaine and control groups^26^. Cocaine HCl also caused changes in relative abundance (increases and decreases) throughout the bacterial taxonomy^24–26^.

While we did not assess alterations in gut function presently, Chivero et al. did show significant cocaine HCl-induced changes in gut function in their study^26^. With their results in mind, we attempted to link drug-induced changes in the gut microbiota to taxa known to alter gut function and inflammation. The LEfSe analysis showed that cocaine HCl was characterized by increases in *Akkermansia municiphila* (genus Verucomicrobia) and Porphyromonadaceae (genus Bacteroidetes). In general, *A. municiphila* has been linked to beneficial roles in gut inflammatory conditions such as high fat diet-induced metabolic problems^59^, alcohol use disorder^60^ and in irritable bowel disease^61^. *A. municiphila* has even been used as a probiotic to improve outcomes in a mouse model of chronic colitis^62^. Lower levels of Porphyromonadaceae have been associated with suppression of inflammation in mice lacking the fat mass and obesity-associated gene^63^. These latter effects indicate that cocaine HCl is exerting inflammatory effects in the gut and the increase in *A. municiphila*, a prominent mucin degrading bacterium^64^, suggests the possibility of enhanced mucin breakdown in support of the findings of Chivero et al.^26^. A similar situation exists for cocaine MI with regard to its signature on the gut microbiota. *Barnesiella* is elevated in Parkinson’s disease patients with cognitive impairment^65^ and is reduced in probiotic-induced improvement in metabolic disorders^66^, suggesting its association with detrimental aspects of these disorders. Lachnospiraceae is increased by high fat diet-induced inflammation^67^ but is decreased in a rodent model^68^ of Parkinson’s disease and in patients with this neurodegenerative disorder^69^ with associated GI problems. These findings suggest the association of Lachnospiraceae with inflammatory conditions or with attempts to mount an anti-inflammatory response, depending on the provoking GI condition. The outcomes of the PICRUSt metabolic and functional analyses were parallel to the drug-induced alterations in the gut microbiome, showing that cocaine HCl significantly increased whereas MDPV resulted in significant reductions in the same metabolic pathways. Overall, cocaine HCl and cocaine MI to a lesser extent increased activity in the major metabolic pathways for nucleotide metabolism, cofactors and vitamins whereas MDPV had the opposite effect on these pathways. Taken together, the results from gut microbiota analyses and the metabolic and functional predictions confirm that cocaine HCl and cocaine MI were very similar whereas MDPV was very different from the cocaine congeners in its effects. This suggests that the treatments drugs are exerting their effects on the gut microbiome directly at the level of the gut and not through an indirect brain-to-gut axis.

If it is assumed that the treatment drugs are acting locally in the gut to alter the microbiome, monoamine transporters emerge as likely mediators that can explain cocaine HCl – cocaine MI similarities and MDPV differences from the cocaine congeners. Cocaine HCl and MDPV are powerful blockers of the DAT and the norepinephrine (NE) transporter (NET)^45,70,71^. Cocaine HCl blocks the serotonin (5-HT) transporter (SERT) whereas MDPV is essentially inactive in blocking 5-HT uptake^45,70,71^. Cocaine MI blocks all three transporters but with much lower potency^72^. All three transporters are expressed in the gut^73–76^ and can influence intestinal motility and function^74,77–80^. Thus, the cocaine congeners would increase extracellular levels of DA, NE and 5-HT whereas MDPV would alter dopamine and NE levels without changing 5-HT. It therefore appears that 5-HT is the factor that differentiates the actions of the cocaine congeners from MDPV with regard to alterations in the gut microbiota.

Despite the similarities of cocaine HCl and MDPV as DAT/NET blockers, it has been revealed recently that their transporter binding kinetics differ significantly and in a manner that impacts function. Sitte and colleagues showed that cocaine HCl dissociates rapidly from the DAT and has short-lived behavioral effects whereas MDPV has significantly slower off-kinetics and exerts long-lasting effects^81^. Therefore, the differences between cocaine HCl and MDPV in terms of the specificity of transporter blockade could be amplified in the gut because of the widely differing duration of their inhibition of monoamine uptake. The overlap in the effects of cocaine HCl^26^ with the specific SERT blocker fluoxetine^82^ on the gut microbiota and GI function (e.g., both increase gut barrier permeability) further substantiates this possibility. Additional studies will be required to determine if blockade of the SERT by cocaine HCl and cocaine MI underlies the currently observed changes by comparison to MDPV.

It is known that cocaine HCl and MDPV share the ability to cause vasoconstriction and increases in blood pressure^83–85^ and that the gut microbiome can regulate blood pressure^86^. However, it does not appear that the cardiovascular effects of these drugs can account for their differing effects on the gut microbiome or metabolome. The ability of cocaine HCl and MDPV to block monoamine transporters in the gut suggests the unrecognized possibility that they could influence pathogen colonization and virulence which are now known to be mediated by 5-HT^87^ and NE^27^. Cocaine is known to be markedly immunomodulatory which can increase susceptibility to infection^88^. Antidepressants, many of which are DAT, NET and SERT blockers, are also known to have antimicrobial effects that could contribute to antibiotic resistance^89–91^, actions that could extend to cocaine HCl and MDPV.

We also hypothesized that cocaine HCl would overlap with cocaine MI in its effects on the gut microbiome and metabolome if their actions on the gut microbiota were peripheral. The cocaine congeners do share extensive overlap in their ability to alter CNS excitation and hyperthermia via blockade of peripheral voltage-gated sodium channels^92–94^. In addition, the conditioned taste aversion properties of cocaine HCl are shared by cocaine MI, an effect that has been attributed in part to peripheral sodium channel inhibition^95^ by these drugs. In animals with prior exposure to cocaine HCl, cocaine MI causes DA^96^ and glutamate^34,97^ release in the nucleus accumbens, reinstates an extinguished cocaine HCl CPP, and rapidly alters the activity of ventral tegmental neurons^98^. Wise and Kiyatkin^35^ have shown convincingly that peripheral interoceptive cues associated with cocaine HCl become conditioned to its central actions, explaining how cocaine MI can exert central effects. Mice treated with cocaine MI in the present experiments were not pre-exposed to cocaine HCl, making it unlikely that cocaine MI was causing effects on the gut microbiota via the CNS.

The current study has several principal strengths. First, it expands existing research on cocaine HCl-gut microbiota interactions and extends it to other drugs with similar pharmacological and structural properties. Second, it establishes that drugs with very similar CNS properties can have distinct effects on the gut microbiota, as observed recently for methamphetamine and synthetic psychoactive cathinone drugs^46^. Third, the results suggest that earlier demonstrations of cocaine effects can now be subjected to reinterpretation in light of the extensive impact of these treatments on the gut microbiota. These include the attenuation of the addictive properties of cocaine HCl with a high fat diet, sodium butyrate (used as a histone deacetylase inhibitor) and with ceftriaxone (to upregulate expression of the neuronal glutamate transporter). Our study also has several primary limitations. First, it did not include measures of drug-induced gut or CNS alterations. Second, the method of drug administration used presently (and in other studies of cocaine HCl-gut microbiota interactions^25,26^) simulated self-administration schedules and dosages versus contingent intravenous intake as used in self-administration studies. This limitation would be difficult to overcome because cocaine MI is not self-administered^96^. It also appears that the pharmacological effects of abused drugs override the method of administration or the behavioral assay used, at least in the case of methamphetamine^99^. Third, we cannot yet determine the mechanism by which the study drugs are altering the structure and composition of the gut microbiota. This will be addressed in future studies.

### Supplementary Information

Supplementary Information.

## Data Availability

Raw data or further methodological information from the current study are available upon reasonable request from the corresponding author.
