# Supplementary material for: Cocaine hydrochloride, cocaine methiodide and methylenedioxypyrovalerone (MDPV) cause distinct alterations in the structure and composition of the gut microbiota
Source: Sci Rep. 2023 Aug 23;13:13754. doi: 10.1038/s41598-023-40892-1 (PMC10447462; doi:10.1038/s41598-023-40892-1)
Supplement: Supplementary file 1 — Supplementary Information. [file 41598_2023_40892_MOESM1_ESM.pdf]

β-diversity index	Comparison	p value
Jaccard	Control vs Coc HCl	0.0061
Jaccard	Control vs Coc MI	0.0207
Jaccard	Control vs MDPV	0.0161
Jaccard	Coc HCl vs Coc MI	ns
Jaccard	Coc HCl vs MDPV	0.0051
Jaccard	Coc MI vs MDPV	0.0364
Bray-Curtis	Control vs Coc HCl	0.002
Bray-Curtis	Control vs Coc MI	0.0139
Bray-Curtis	Control vs MDPV	0.0141
Bray-Curtis	Coc HCl vs Coc MI	0.0199
Bray-Curtis	Coc HCl vs MDPV	0.002
Bray-Curtis	Coc MI vs MDPV	0.002

Supplemental Table 1. Pairwise statistical comparisons of treatment drug effects on β-diversity. Abbreviations are Coc HCl (cocaine HCl); Coc MI (cocaine methiodide); MDPV (methylenedioxypyrovalerone).

MetaCyc pathway	Pathway function	Drug-induced alterations in pathway
7208	Xenobiotics biodegradation and metabolism	MDPV>MI; HCl<MDPV; MI<Cont
5121	Metabolism of terpenoids and polyketides	MDPV>HCl; MDPV>MI; HCl<Cont; MI<Cont
7560		MDPV>HCl; MDPV>MI; HCl<Cont
NonmevIPP		MDPV>HCl; MDPV>MI; HCl<Cont
7228	Metabolism of cofactors and vitamins	HCl>MDPV; MDPV<Cont; MI>MDPV
5897		MI>MDPV; HCl>MDPV;, HCl>Cont; MDPV<Cont
5899		MI>MDPV; HCl>MDPV; HCl>Cont; MDPV<Cont
5840		MI>MDPV; HCl>MDPV; HCl>Cont; MDPV<Cont
5845		MI>MDPV; MI>Cont
5862		MI>MDPV; MI>Cont
5861		MI>MDPV; HCl>MDPV; HCl>Cont; MDPV<Cont
5896		MI>MDPV; MI>Cont
5838		MI>MDPV; HCl>MDPV; HCl>Cont; MDPV<Cont
5837		HCl>MDPV; MI>MDPV; HCl>Cont
5850		MI>MDPV; MI>Cont
1CMET2		MDPV>HCl; HCl<Cont; MDPV>MI
7374		HCl<Cont; MI<Cont
5863		HCl>MDPV; MI>MDPV; HCl>Cont
5860		MI>MDPV; MI>Cont
5898		MI>MDPV; HCl>MDPV; HCl>Cont; MDPV<Cont
0-1415		MI>MDPV
Pyridmucsal		MDPV>HCl; MDPV>MI
6385	Glycan biosynthesis and metabolism	MDPV>HCl; MDPV>MI; MI<Cont; HCl<Cont
6386		MDPV>MI; MDPV>HCl; MI<Cont; HCl<Cont
0-1261		MI>MDPV; MDPV<Cont
Galacturocat		MI>Cont; MDPV>Cont
5345	Amino acid metabolism	HCl>MDPV; HCl>Cont
Sulfate-Cys		HCl>MDPV; HCl>Cont
6151		MDPV>Cont; MI>Cont
ASPASN		MDPV>HCl; MDPV>MI
2942		MDPV>HCl; MDPV>MI
Ser-glysyn		MDPV>HCl; MDPV>MI
Thre-syn		MDPV>HCl
tRNA charging		MDPV>MI; MDPV>HCl
7197	Nucleotide metabolism	HCl>MDPV; MDPV<Cont
7184		HCl>MDPV; MDPV<Cont
7196		HCl>MDPV; MDPV<Cont
7187		HCl>MDPV; MDPV<Cont
6277		MDPV>MI; MDPV>HCl; MI<Cont; HCl<Cont
6353		MI>Cont
0-166		HCl>MDPV; MDPV<Cont
Denovopurine		HCl>MDPV; MDPC<Cont; MI>MDPV
0-162		HCl>MDPV; MDPV<Cont; MI>MDPV
6123		MDPV>HCl; MDPV>MI: HCl<Cont
6125		HCl>MDPV; MDPV<Cont; MI>MDPV
6122		MDPV>MI; MDPV>HCl; MI<Cont; HCl<Cont
7200		HCl>MDPV; MDPV<Cont

7211		HCl>MDPV; MDPV<Cont; MI>MDPV
841		HCl>MDPV; MDPV<Cont; MI>MDPV
6121		MDPV>MI; MDPV>HCl; MI<Cont; HCl<Cont
6545		HCl>MDPV
6609		MDPV>HCl; MDPV>MI
7219		MDPV>MI; MDPV>HCl
7221		MDPV>HCl; MDPV>MI
5973	Lipid metabolism	MDPV>MI; MDPV>HCl; HCl<Cont; MI<Cont
7663		MDPV>HCl; MDPV>MI; HCl<Cont; MI<Cont
Golpdlcat		HCl>Cont; HCl>MDPV; HCl>MI
SO4ASSIM	Energy metabolism	HCl>MDPV; HCl>Cont
CODH		MI>MDPV; MDPV<Cont
TCA		HCl>MDPV; MI>MDPV; MDPV<Cont
6969	Carbohydrate metabolism	HCl>MDPC; MDPV<Cont; MI>MDPV
FUCCAT		HCl>Cont; MI>Cont
7242		MI>Cont; MDPV>Cont
5913		HCl>MDPV; MI>MDPV; MDPV<Cont
1061		HCl>MDPV; MI>MDPV; HCl>Cont
Glucose-1 PO4		HCl>MDPV; HCl>Cont; MI>MDPV
6749		MDPV>Cont; MI>Cont; HCl>Cont
COLANSYN		HCl>MDPV
Gluconeo		MDPV>HCl
Glycogensyn		MDPV>MI; MDPV>HCl
0-1241		HCl>MDPV
5100		MDPV>HCl; MDPV>MI
Polyaminesyn3	Amine and polyamine metabolism	MI>MDPV; HCl>MDPV
Pepidoglycansyn	Cell structure metabolism	MDPV>MI; MDPV>HCl
6387		MDPV>MI; MDPV>HCl
6163	Aromatic compound metabolism	MDPV>MI; MDPV>HCl

Supplemental Table 2. Effects of treatment drugs on inferred metabolomic alterations from PiCRUST analysis. Treatment abbreviations are HCl (cocaine HCl); MI (cocaine MI); Cont (control). All treatment effects were statistically significant with p values ranging from < 0.001 to < 0.05.

MetaCyc pathway	Coc HCl	Coc MI	MDPV
5121	↓	↓	no change
5973	↓	↓	no change
6121	↓	↓	no change
6122	↓	↓	no change
6749	↑	↑	no change
6277	↓	↓	no change
FUCCAT	↑	↑	no change
6385	↓	↓	no change
6386	↓	↓	no change
7663	↓	↓	no change
5899	↑	no change	↓
5898	↑	no change	↓
5861	↑	no change	↓
5840	↑	no change	↓
5897	↑	no change	↓
5838	↑	No change	↓

Supplemental Table 3. Shared metabolic pathway alterations by Coc-HCl and Coc MI and by Coc HCl and MDPV. Abbreviations are coc HCl (cocaine HCl); coc MI (cocaine MI); MDPV (Methylenedioxypyrovalerone). All changes were by comparison to control and were statistically significant with p values ranging from < 0.001 to < 0.05.
